# Uses and Abuses of Patient Reported Outcome Measures (PROMs): Potential Iatrogenic Impact of PROMs Implementation and How It Can Be Mitigated

**DOI:** 10.1007/s10488-013-0509-1

**Published:** 2013-07-19

**Authors:** Miranda Wolpert

**Affiliations:** Child and Adolescent Mental Health Services Evidence Based Practice Unit (CAMHS EBPU), UCL and Anna Freud Centre, 21 Maresfield Gardens, London, NW3 5SU UK

**Keywords:** PROMS, Service evaluation, Outcome measures, Patient reported outcome measures, Clinical tools

## Abstract

Having been a national advocate for the use of patient reported outcome measures (PROMs) in Child and Adolescent Mental Health Services (CAMHS) in the UK for the last decade, I have become increasingly concerned that unless the potential iatrogenic impact of widespread policy requirement for use of PROMs (Department of Health, Children and Young People’s Health Outcomes Strategy, 2012) is recognised and addressed their real potential benefits (Sapyta et al., J Clin Psychol 61(2):145–153, 2005) may never be realized. Drawing on examples from PROMs implementation in CAMHS in the UK (Wolpert et al., J Ment Health 21(2):165–173, 2012a; Child Adolesc Mental Health 17(3):129–130, 2012b). I suggest key ways forward if PROMs are to support best clinical practice rather than undermine it.

## What Are PROMs?

Patient reported outcome measures (PROMs) refer to any questionnaire completed by those using services (in the case of child mental health services this includes parents and carers as well as children and young people) that is used to try to assess whether there has been improvement in one or more domains relevant to the outcome of treatment. Thus PROMs may, for example, measure change in symptoms or impact of difficulties on the young person’s life and/or sense of wellbeing. PROMs should be distinguished from clinician rated outcome measures (CROMs) which are clinician completed questionnaires relevant to assessing treatment outcomes. PROMs should also be distinguished from patient reported experience measures (PREMs) which measure the patient’s satisfaction with a service they received but not the “outcome” of the service as such.

## What Are PROMs For?

Many PROMs were originally designed as epidemiological tools to identify patterns of symptomatology or wellbeing. They were then used as pre and post measures to try to evaluate the impact of interventions as part of controlled or naturalistic studies. It was only following their use as tools for research and evaluation that there became a call for PROMs to be used by individual practitioners to enhance the clinical management of individual patients as part of feedback systems (Bickman 2008; Black 2013).

I would argue that there is a tension between these two overarching aims; to collect data to inform generalizable findings including audit and research on the one hand, versus the desire to collect data to inform individual care on the other. By conflating these two aims we may fail to put in place appropriate structures to ensure that the particular challenges raised by each, as well as the tensions between them, do not undermine both.

## How Do PROMs Work for Audit and Research Purposes?

PROMs use to inform audit and research involves data being collected, aggregated and analysed at a system level (Department of Health 2012; Devlin et al. 2010). The tools need to be psychometrically robust and the data need to be as complete as possible to prevent false interpretation (Clark et al. 2008). These sort of data made public and shared within careful parameters (Black 2013; Spiegelhalter 2005) have been shown to powerfully influence improvements in service quality and outcomes in a range of specialities (Porter 2010). Making such data available and making use of it for quality control is at the heart of the attempts to improve quality across state funded health systems such as in the UK (Department of Health 2012; Francis 2013).

The aspiration is that aggregated data will in time inform direct clinical care by allowing clinicians to identify and consider differences in outcomes between individuals in their care and appropriate group norms, though this requires careful modelling of a sort still in its infancy (Lutz et al. 2007).

## What Are the Potential Iatrogenic Consequences of the Use of PROMs for Audit and Research Purposes?

The benefits of using PROMs for audit or research can feel quite distal from the daily dilemmas and decision making challenges facing those implementing them on the ground and can feel separate from, and even undermining of, the clinical encounter. The standard questions may seem irrelevant to a given patient and can be experienced as a potential burden for clinicians and patients alike and raise anxieties about use to limit service provision (Moran et al. 2012). Clinicians in particular can experience PROMs in this context as an additional bureaucratic burden, imposed autocratically from above, particularly in the context of lack of adequate IT to support their use in a non-resource intensive way and escalating demands from managers for more and more form filling (Batty et al. 2012).

As part of the CAMHS Outcomes Research Consortium (CORC), a learning collaboration of Child and Adolescent Mental Health Services (CAMHS) across the UK and Europe, committed to using PROMs to inform service improvement, I and others have been instrumental in recommending use of key measures such as the Strengths and Difficulties Questionnaire to assess patients’ wellbeing and symptoms at the start and outcome of treatment. In part this is because such measures had access to national norms and thus could potentially be used to assess the “added value” of service intervention (Ford et al. 2009). What we have discovered in practice in the UK is that this has meant national funders of services mandating the use of this measure for services, setting targets for completion rates and that little attention has been paid to its integration with clinical conversations or clinical care. This, combined with clinician anxiety and concern over measure use, has led to a situation where clinicians across the UK may never see the completed questionnaires in time to use them in sessions with patients and service users never get to hear what their scores mean or how they are used, which may severely limit potential positive benefits (de Jong et al. 2012).

## How Should PROMs Be Implemented for Research and Audit in Such a Way as to Mitigate Potential Iatrogenic Impact?

Whilst clinicians should be encouraged to collect PROMs data to inform national aggregation, trained in how to implement and challenged if they argue they feel such an approach is never helpful, ultimately there may need to be at least some freedom for clinical judgment in relation to PROMs use. Whilst there is no evidence of actual harm caused by use of PROMs and rather more evidence of anxiety about use of PROMs inhibiting use (Batty et al. 2012) there is emerging evidence that intensive PROMs may have a less positive impact in certain contexts such as in inpatient services or with young adults in crisis (de Jong et al. 2012; Vane Oenen personal communication). It may be important to be more explicit in roll out of PROMs nationally about how new an approach this is and how little we know about the psychometric properties, impact or indeed utility of many of the measures being used.

Any targets in relation to PROMs use should be related to stage of implementation of PROMs (for example whether a service has just started to use PROMs) and should concentrate on clinical use of data to inform practice, rather than assessing success of implementation in terms of how much data has been collected for central analysis (CAMHS Outcomes Research Consortium 2013).

It is important that data is aggregated and fed-back rapidly but also in ways that are appropriate to the flaws and tentativeness of the data (Spiegelhalter 2005). All those wishing to use these data should be encouraged to appreciate that PROMs data alone are unlikely to be able to yield reliable results and will need to be triangulated with other data sources. For example at the level of service evaluation consideration will need to be given to case mix variables, staffing variables and other indicators of quality such as level of complaints, drop out rates and referrer satisfaction. Furthermore, data should be interpreted in relation to underlying theories of processes and mechanisms.

## How Do PROMs Work as Clinical Tools?

PROMs as clinical tools need to be sensitive to the situation of the individual patients and able to provide insights that can inform direct clinical decision-making and enhance experiences of care. They can be conceived of as providers of feedback and tools to monitor for change and in this regard need to be distinguished from those PROMs that may only be used, for example, to consider impact after an episode of care is complete (Glasziou et al. 2008; Sapyta et al. 2005).

Use of PROMs as clinical tools in mental health settings has been shown to improve experiences and outcomes for people at risk of treatment failure (Bickman et al. 2011; Whipple and Lambert 2011). They are being promoted as ways to support and enhance increasingly collaborative models of patient-clinician interaction and shared decision making (Coulter 2010) and to help ensure service users voices are heard (Greenhalgh 2009; Lutz et al. 2007; Marshall et al. 2005), particularly in the context of work with children and young people or other groups dependent on carers to allow access to services (Curtis-Tyler 2011).

Children and Young People’s Improving Access to Psychological Therapies (CYP IAPT), a national UK initiative to “transform CAMHS” currently being rolled out to around 60 % of the country, has at its heart a commitment to implementation of PROMs and there is feedback from clinicians and service users involved of the use of the approach recommended to directly inform their clinical work: “It means if we go off track or get a bit lost along the way, we can both figure out how to find the way back again.” Young person from YoungMinds’ Very Important Kids Group reporting on experience of the CYP IAPT mode” (O’Herlihy 2013; Porter 2010 p. 3).

The approach includes both standardised and idiographic measures and is supported by service users (Badham 2011). Initial feedback suggests impacts on patient–clinician interaction in terms of helping develop more transparent and collaborative ways of working, though challenges remain about the burden of administration and data capture (Porter 2010).

## What Might Be the Iatrogenic Consequences of the Use of PROMs for Direct Clinical Work?

Whilst many clinicians and patients are supportive of the use of PROMs to help monitor progress and enhance communication, both groups have expressed concerns about instrument validity, time and support necessary for implementation and that instruments may generate information that could be used in ways that disadvantage patients or to limit access to services (Badham 2011; Curtis-Tyler 2011; de Jong et al. 2012; Moran et al. 2012; O’Herlihy 2013).

CYP IAPT has been concerned from the start to support use of PROMs both for audit purposes and for direct clinical use and offers a suite of PROMs for clinicians to choose from (Wolpert et al. 2012a; b) stressing the need for clinical judgement to be used in selection. It has emerged that some Provider Organisations have mandated certain PROMs be used in all cases regardless of their clinical utility and have set targets and rewards for use that clinicians experience as not taking into account context for particular groups or clinical need.

## How Should PROMs Be Used and Interpreted in Terms of Direct Clinical Care to Mitigate Potential Iatrogenic Impact?

It may be crucial that in introducing PROMs into clinical practice front line clinicians are introduced to the tools through the prism of collaborative working and shared decision making rather than as tools primarily used for audit or performance review. The current author together with colleagues has developed the Using Patient Reported Outcome Measures to Improve Service Effectiveness (UPROMISE) training approach, currently being trialed across the UK with NHS and voluntary sector providers of CAMHS. Our learning from this is that an underlying ethos of collaborative working and shared decision making, and a focus on using PROMs as part of clinical conversations, promotes greater clinician engagement and willingness to trial the use of PROMs. Videos of PROMs use (e.g. http://www.corc.uk.net/resources/training-resources) can be particularly beneficial in supporting early implementation. It may also be helpful to explore the different ways PROMs data can be used directly with patients.

It is going to be increasingly important that all frontline clinicians, managers, commissioners and board members become skilled in use of sophisticated statistical process control methods and aware of the dangers of over interpretation of random fluctuations due to measurement error or chance movement (Glasziou et al. 2009). Concepts such as reliable change indices, differences in effect sizes, use of process and control charts, and their limitations in healthcare settings, need to become widespread currency across all disciplines and areas using PROMs and our early attempts to provide such training have been well received (Childs 2013). One analogy I have found useful in teaching front line practitioners in CAMHS about use of PROMs is to compare them to measuring someone’s height but your hand is shaking. The more sensitive the PROMs the less shaky the hand, but all that I know of currently are pretty shaky and many in my field involve great swoops of the hand up and down.

## Conclusions

The UK is in the process of a major experiment in terms of rolling out a new form of intervention—use of PROMs—but we are doing so currently without having trained people in their use. This is potentially extremely dangerous. If we replaced the word PROMs with “taking blood” we might be concerned to learn this was being widely mandated without clinicians knowing the answers to key questions such as: how best to safely interpret and report the data; how often to use in clinical practice; how best to introduce; how much change is enough and when not to use.

**Box 1:** Key messages

What is urgently required for PROMs to both inform research and audit and to support clinical practice:
Explicit recognition of need to disaggregate two aims—use of PROMs for research and audit versus use for direct clinical care.
Training for front line clinicians in how to introduce, input, score and interpret PROMs in context of collaborative working.
Training for service managers, board members, commissioners and others in how to interpret scores and what the limitations are to are to their use without further triangulation.
Further research into PROMs use in clinical practice: how best to safely interpret and report the data: how often to use in clinical practice; how best to introduce; how much change is enough; when not to use.
